# Time-resolved monitoring of biofouling development on a flat sheet membrane using optical coherence tomography

**DOI:** 10.1038/s41598-017-00051-9

**Published:** 2017-02-07

**Authors:** Luca Fortunato, Sanghyun Jeong, TorOve Leiknes

**Affiliations:** Water Desalination and Reuse Center (WDRC), Biological and Environmental Science & Engineering (BESE), King Abdullah University of Science and Technology (KAUST), Thuwal, 23955-6900 Saudi Arabia

## Abstract

Biofouling on a membrane leads to significant performance decrease in filtration processes. In this study, an optical coherence tomography (OCT) was used to perform a time-resolved analysis of dynamic biofouling development on a submerged membrane under continuous operation. A real-time change in the biofouling morphology was calculated through the image analysis of OCT scans. Three videos were generated through the acquisition of serial static images. This is the first study that displays the dynamic biofouling formation process as a video. The acquisition of OCT cross-sectional scans of the biofouling allowed to evaluate the time-lapsed evolution for three different time periods (early stage, double layers and long-term). Firstly, at the early filtration stage, membrane coverage and average biofouling layer thickness were found to be linearly correlated with the permeate flux pattern. Secondly, after 3 d of operation, an anomalous morphology was observed, constituted by a double-layered biofouling structure: denser on the bottom and looser on the top. In a long-term operation, the biofouling structure underwent a dynamic evolution over time, resulting in a multi-layered structure. The biofouling formation information was closely associated with filtration performance (i.e. flux) indicating the suitability of OCT as real-time and *in-situ* biofouling monitoring technique.

## Introduction

The growth of a fouling layer by deposition of undesirable materials such as suspended solids, colloids and microbial cells onto or into a membrane is a persistent problem in membrane filtration processes. Bacterial accumulation on the membrane can occur through two processes: attachment (bioadhesion and bioadsorption) and growth (multiplication). Extracellular polymeric substances (EPS) constitute the largest fraction (50–80% of the total organic matter and protein) of the biofilm composition^1^. In particular, biofilm formation causes an unacceptable decline in membrane performance. The performance losses involve an increase in feed channel pressure drop, permeate flux decline, and rejection reduction^2^. Recently, low-pressure driven membrane processes as microfiltration (MF) and ultrafiltration (UF), employed mainly in a submerged configuration, are increasingly used in advanced water treatment^3–5^. In a submerged membrane bioreactor (SMBR), the biomass accumulation (or biofouling) on the membrane surface is severe as the purpose of the membrane is to retain the biomass in the reactor. Although there are various factors that affect membrane fouling of MBR, such as membrane properties, biomass properties, feedwater characteristics and operating conditions, membrane biofouling via microbial products plays a critical role in determining the MBR performance^6^. This causes an increase in transmembrane pressure (TMP) under constant flux operation or a decrease in the permeate flux under constant pressure mode. Although flux decline or TMP increase are good indicators of biofouling, they do not provide reliable information for the extent of pore blocking or the thickness of a cake layer on the membrane, information necessary to comprehend the mechanism and nature of the membrane biofouling.

A detailed biofouling characterization in membrane-based water treatment processes is crucial to the improvement of a filtration performance, as well as to the establishment of a fouling prevention strategy. Various observation techniques have been extensively applied to study the biofouling accumulation in membrane filtration systems^7^. *Ex-situ* methods (i.e. membrane autopsies), wherein a fouled membrane is taken out of the system after operation, can provide important insights to the thickness and structure of the biofouling on membrane^8, 9^. However, these approaches can give misleading results as biofilms or biofouling layers could change in nature due the manor in which samples are collected, and only characterize conditions representative of the point of time point at which filtration was terminated. Furthermore, sample analysis typically involves destructive procedures.

Severe flux decline and process failure are usually observed if the produced water quality shows under a given set of standards. However, the water quality analysis may be the most expensive method for fouling monitoring and cannot provide any structural information about fouling, which is required to establish the biofouling preventing strategy, but is still widely adopted due to its practicality. Membrane biofouling is a very dynamic process, which implies that formation of a biofouling layer is a function of filtration time, and the focus lately has therefore been on *in situ*, and online (or real-time) monitoring techniques. Several *in situ* monitoring techniques have been developed in the laboratory to reveal the growth and behavior of biofouling layers in membrane filtration processes^10, 11^.

Optical coherence tomography (OCT), an emerging imaging technique mainly used in biomedical applications^12, 13^, has recently gained popularity in the study of biofouling in membrane filtration processes. OCT is a non-destructive technique that uses backscattered light to produce images of the biofouling layer and is capable of acquiring real-time dynamic cross-sectional images of the fouling layer at millimeter scale. Derlon *et al.* ^3^ applied the OCT for offline autopsy of membrane coupons. Gao *et al.* ^14^ characterized the fouling layer formed by the deposition of bentonite micro-particles in a filtration cell. The OCT was recently used to assess the biofouling spatial distribution in spacer filled channel^15^. OCT time-lapse measurements have been reported and used in the evaluation the impact of shear stress conditions on biofilm behavior^16^.

In the present study, the OCT technique was employed to provide a real-time, and cross-sectional profiling of biofouling layer property changes (i.e. thickness) in a gravity-driven submerged membrane bioreactor (GD-SMBR). The objective of this work is to evaluate the effectiveness of time-lapse resolved image analysis in the study of the dynamic development (or structural changes during biofilm growth) of the biofouling formed in membrane filtration processes under continuous operation. This time-lapse monitoring of the dynamic evolution is expected to provide a breakthrough in the understanding of the biofouling formation and structural changes on the submerged membrane.

## Results and Discussion

In the present work, OCT enabled continuous and non-destructive monitoring of the biofouling development on the submerged flat sheet membrane. The dynamic formation of the biofouling layer was investigated through two experiments. OCT cross-sectional scans were acquired at different frame rates. The time-resolved images analysis provided detailed information on the dynamic formation of the biofouling layer, showing that a decrease in flux during the early stages of operation could be directly correlated with the biofilm development. The OCT scans show how the biofilm evolves over time forming a double-layered morphology after 3 d of operation before establishing a multi-layer structure that appears to stabilize over the time. At the end of the experimental period a sample was imaged using ESEM, confirming the structure results of the biofouling layer observed non-destructively with OCT. Results indicate that there are clearly distinct phases from the initiation of the filtration processes until a steady state is observed.

### Early attachment

Fouling in MBR processes is very complicated and the result of different causes^17, 18^. When the foulant size is comparable or smaller than the membrane pores, pore blocking may occur, while cake layer formation on the membrane surface tends to dominate when the foulant size is larger. Lee *et al.* ^19^ reported that cake layer formation is the main cause of fouling in UF membranes, representing around 80% of the total hydraulic resistance.

Recently, several techniques have been introduced to monitor fouling non-destructively and determine cake layer thickness as function of the time. Electrical impedance spectroscopy (EIS) was used to monitor on-line the fouling in reverse osmosis system by making the correlation to the buildup of a foulant layer on the membrane surface^20^. Ultrasonic time-domain reflectometry (UTDR) was employed to measure the fouling layer deposited in crossflow cell^21^ and in submerged hollow fiber membrane module to measure the relationship between the operational flux and particle deposition on the membrane surface^22^. In the present study, OCT was used to investigate the impact of early stage biofouling formation over a flat-sheet membrane and how it affected the permeate flux. Cross sectional scans of the membrane were acquired with a frequency of 10 min after 12 h of operation, aiming to have a better understanding of the initial behavior of the biofouling formation. It must be acknowledged that this is the first attempt to monitor the early stage of the biofouling development directly in the system in real-time and non-destructively under continuous operation (with a rapid scan frequency), without the use of any staining or contrast agent.

**Figure 1:**
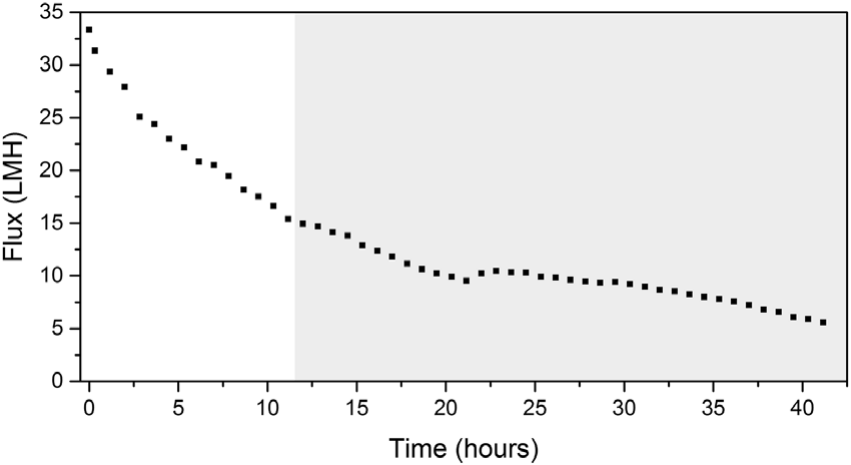
Flux decrease during the first 42 h of filtration time (Observation period by Supplementary Video 1 is highlighted in grey color).

**Figure 2:**
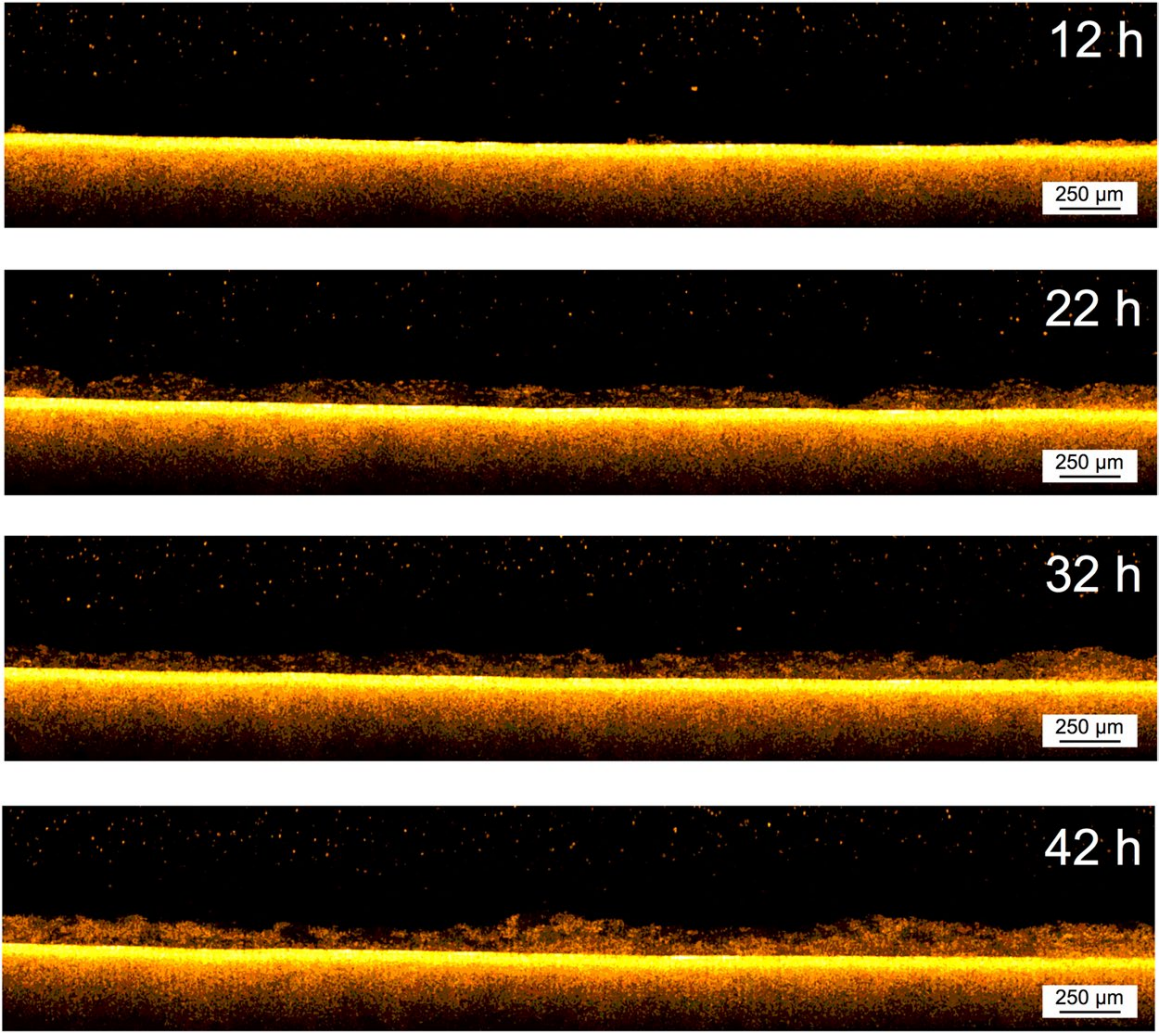
A time-lapse development of early deposition of biomass on a flat sheet membrane in GD-SMBR (from 12 to 42 h) (See Supplementary Video 1).

**Figure 3:**
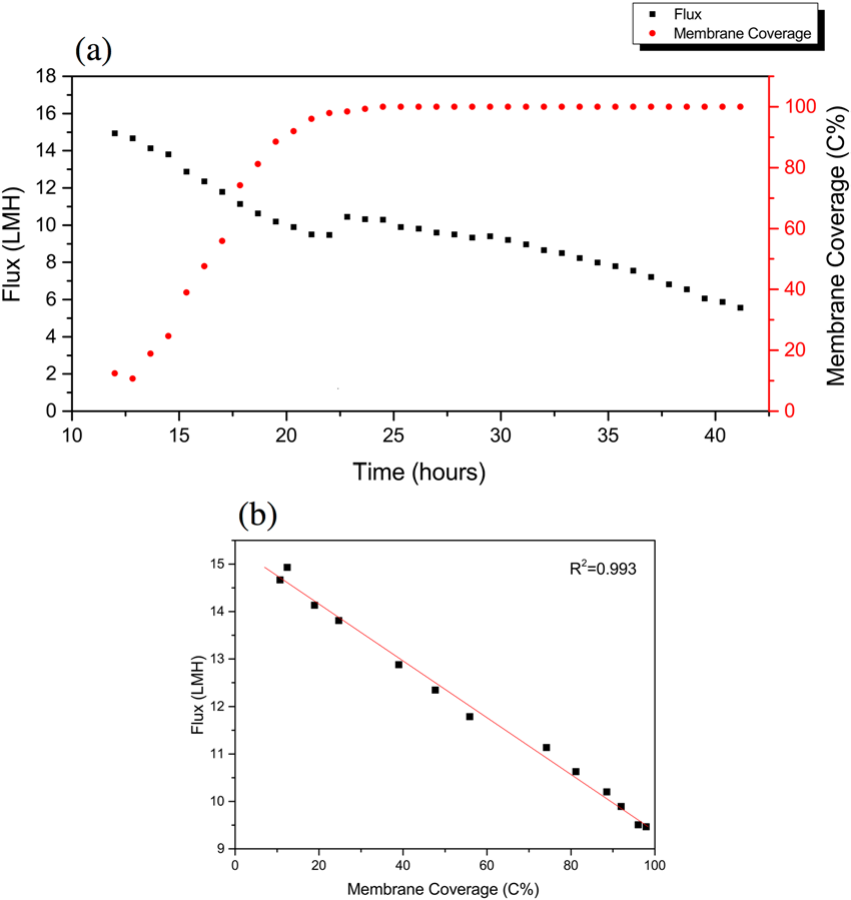
(**a**) The relationship between membrane coverage and permeate flux in the function of filtration time. (**b**) Correlation between membrane coverage and permeate flux (Phase I: from 12 to 22 h).

**Figure 4:**
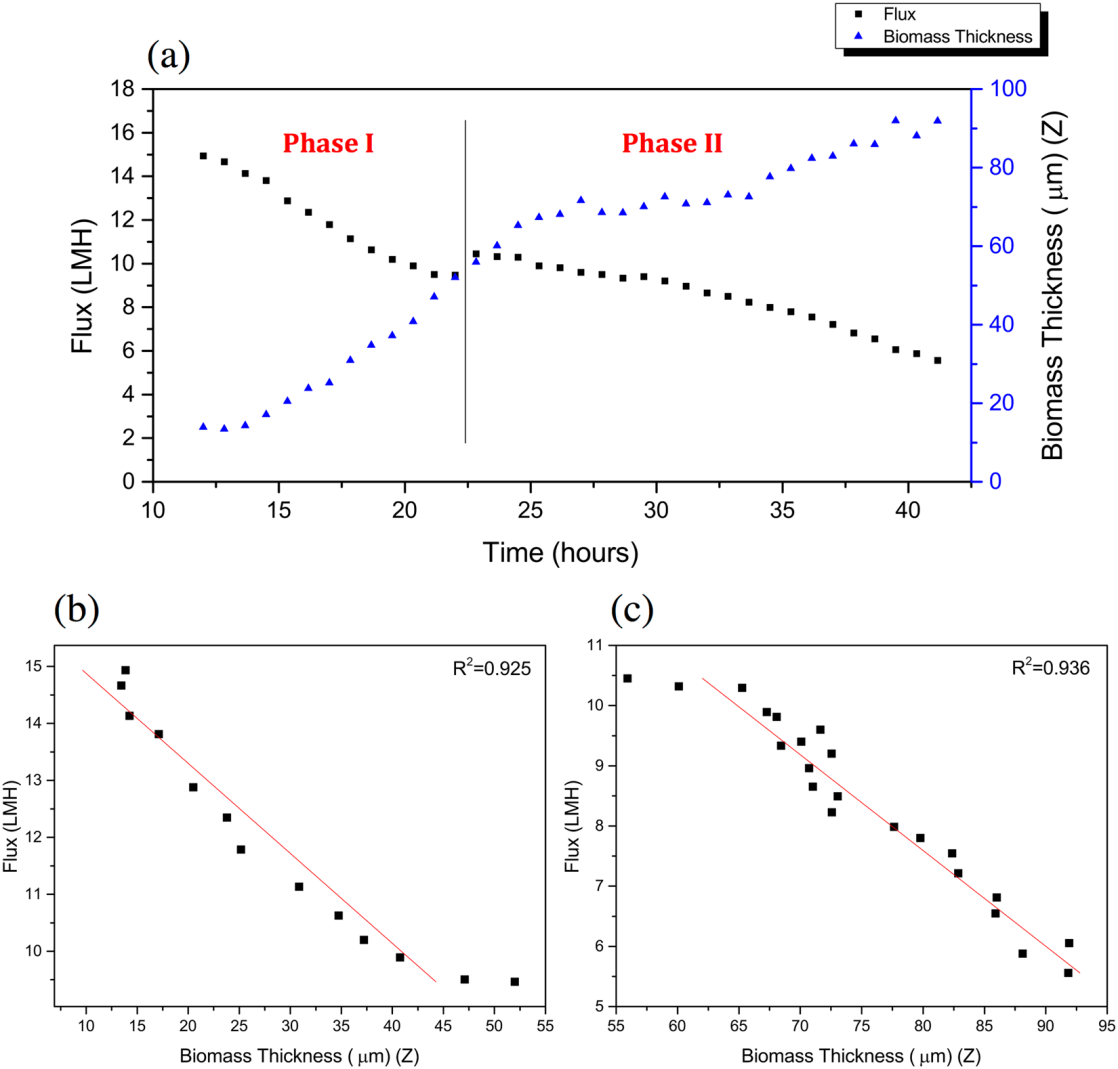
(**a**) The relationship between average biofouling layer thickness and flux in the function of time (the line divides the two phases). (**b**) Correlation between average biofouling layer thickness and permeate flux in the phase I (from 12 to 22 h). (**c**) Correlation between average biofouling layer thickness and permeate flux in the phase II (from 23 to 42 h).

As stated by Wang^23^, the permeate flux decrease during the earlier stage is mainly due to the pore blocking mechanism. As shown in Fig. 1, the flux decline in the early stage (from 0 to 12 h) was greater compared to the rate of decline in the later stages. The on-line cross-sectional investigation performed on the membrane was in agreement with the flux decrease. As shown in the beginning of the Supplementary Video 1 and Fig. 2 (at 12 h of operation), as the initial flux decline occurs only a small amount of biomass is observed on the membrane surface. Hence, as shown by the OCT scans, the initial rapid flux decrease cannot be attributed to the formation of a cake layer, suggesting that pore blocking is dominated. Compared to other imaging techniques (i.e. confocal laser scanning microscopy and scanning electron microscope) the OCT is limited by a lower resolution and higher noise (speckle). It must be noted that the OCT is only capable of detecting particles in the micrometers range. In this study a 20 KDa membrane (pore size ≈ 0.01 μm) was used and consequently pore blocking will occur due to the smaller foulants present in the water. In the first 12 h of operation when the flux decreased from 33.3 to 14.9 L/m^2^h (Fig. 1), no increase in biofouling was observed (Fig. 2). An increase in biomass deposition on the membrane is started to observe after 12 h. From 12 to 42 h (Fig. 2 and Supplementary Video 1) the membrane performance in terms of flux decline, can be directly related to the deposition of biofouling and cake layer formation over the membrane surface. Combining the data from analysis of the OCT scans and measured flux decrease is possible to define different steps or phases in the early stages of filtration of this study. In order to relate the membrane performance to the biofouling morphology, membrane coverage and average biofouling layer thickness, these values are calculated from the acquired scans (Eqs 1–3 in the supplementary information). The flux decline in the period from 12 to 22 h can be linearly correlated with the membrane coverage (Fig. 3a and b). When the biofouling covered the majority of the area observed (i.e. 98% of membrane coverage), a small jump in the flux was observed (Fig. 3a). This is attributed to a possible change in dominant fouling mechanism or a change in filtration performance (i.e. permeate quality). Analyzing the average biofouling layer thickness and membrane coverage of the deposited biomass as a function of the flux trend, two different zones or phases are apparent in the early stages of filtration (from 12 to 42 h), shown in Fig. 4a. In the initial phase (phase I: from 12 to 22 h), the biomass does not cover the whole membrane surface and the biofouling layer thickness and membrane coverage increase linearly. In the second stage (phase II: from 23 to 42 h), the biomass covers the whole surface and an increase in an average thickness of biofouling layer was observed. As presented in Fig. 4b and c, a linear correlation between average thickness and the flux was observed before and after the whole coverage of the monitored area (phases I and II). Independent experiments were performed to support this finding (see supplementary information). Therefore, thanks to the use of time-resolved monitoring and image analysis, it was possible to study the effect of early biofouling formation on the filtration performance of a GD-SMBR. The flux measurements in combination with assessing membrane coverage and average biofouling layer thickness from OCT image analysis, allows distinguishing the different behavior of the filtration process in then early stages. In summary, when the membrane is not fully covered by biomass, the flux decreases linearly as a function of biomass deposition on the membrane surface (i.e. membrane coverage), while once the biomass fully covers the whole area monitored the flux decreases linearly as a function of increase of the biofouling layer thickness. Moreover, the on-line and non-destructive biofouling information (or biofouling descriptor) could be useful to validate the filtration mechanism and evaluate the impact of biofouling formation on the flux decrease.

### Double layer structure

**Figure 5:**
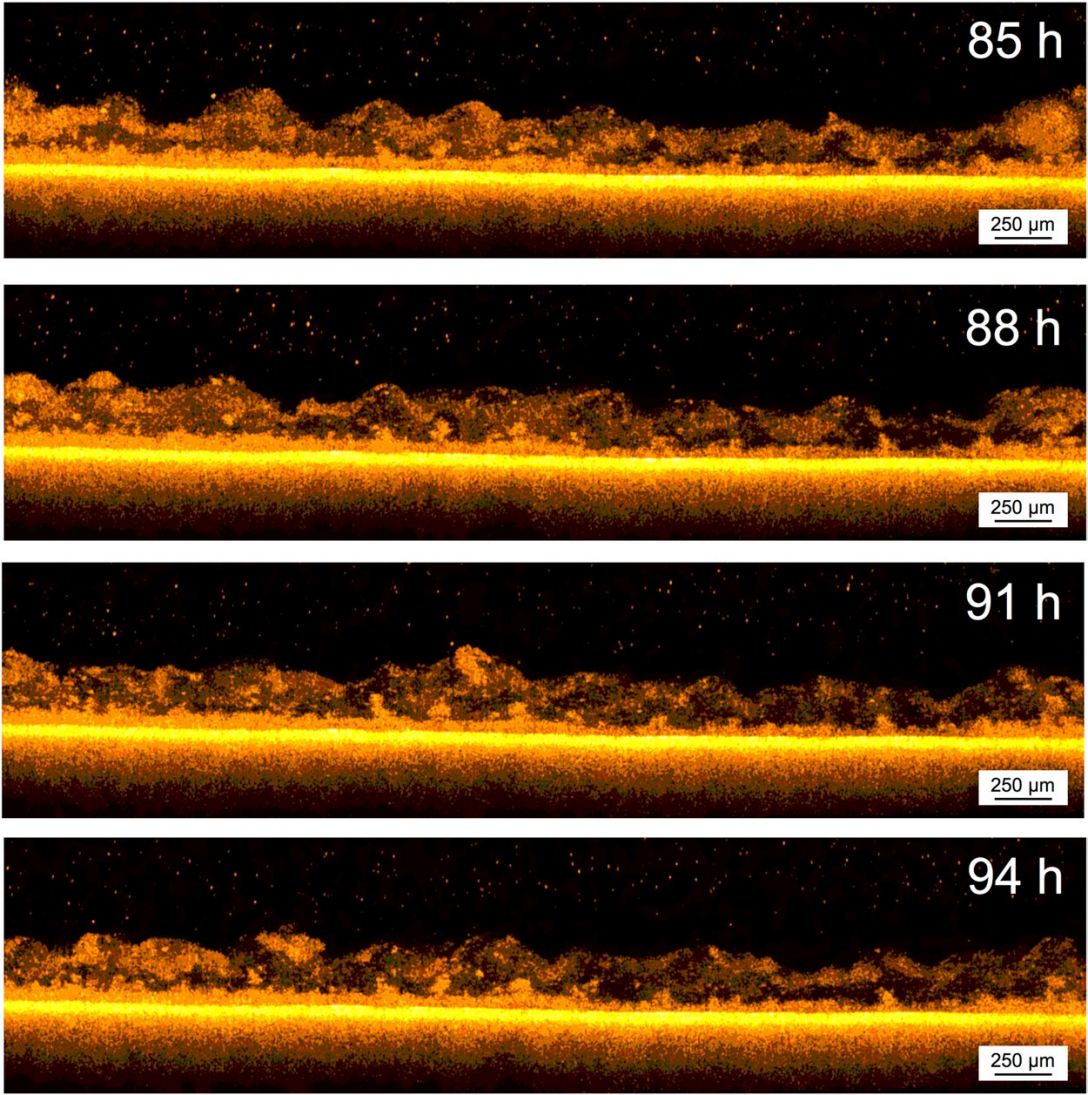
OCT cross-sectional scans of the double layer biofouling morphology. The scans were acquired with a frequency of 5 min from 84 to 96 h of the experiment. The lower layer remains constant while the upper one moves (See Supplementary Video 2).

**Figure 6:**
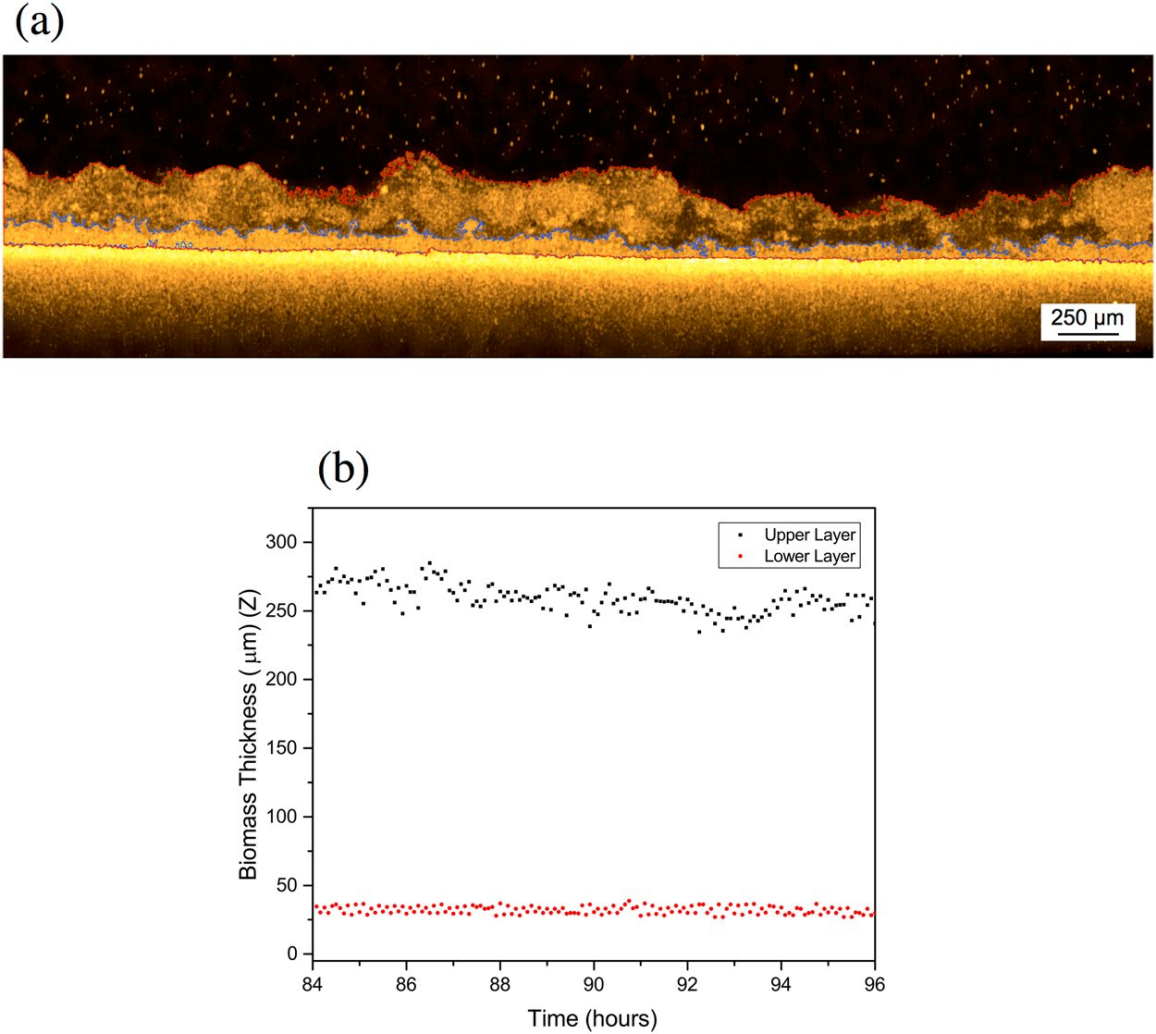
(**a**) Morphology observed after 84 h non-destructively and directly in the filtration tank with OCT. The biofouling presents a double-layered structure. The lines in red and in blue delimit the upper and lower layer, respectively. (**b**) Average thickness of the double layers over the time. The structure was monitored for a period of 12 h (OCT scans acquired every 5 min) after 84 h from the beginning of the experiment.

As reported by Akkhondi *et al.* ^5^ the permeate flux decrease pattern was divided into four different phases under gravity driven operation during long-term filtration^24^. In addition, the phases were related to a change in the biofouling morphology. In the present work, the first day of operation was investigated in detail (from 84 to 96 h), acquiring images for 12 h with a scans frequency of 5 min after 3 d of operation (Table S1), with a corresponding video composed of 144 frames (Supplementary Video 2). As can be seen from OCT scans (Fig. 5) and the Supplementary Video 2, an anomalous structure was observed. The biofouling formed on the membrane is clearly observed as two different layers; a lower dense and compact layer, and an upper loose and thicker one (Fig. 6a). A similar morphology was also reported in a previous paper by Blauert *et al.* ^16^. The difference between these two layers is the thickness as well as the macro-porosity, where the OCT scans show that the upper layer appears as a “cloud” above lower layer.

This anomalous morphology could be a trait of the initial phase of the biofouling formation and transitions as the biomass acclimates to the system. This particular morphology may also be linked to the hydrodynamic condition of the system. In this study, GD-SMBR was only operated under gravity with a corresponding pressure of 4.5 kPa, which is in an ultralow pressure range. The upper part of the loose layer can be seen to move while the lower one appears to be stationary, and maintaining an average thickness (Fig. 6b). As evidently seen from the Supplementary Video 2, the upper layer also appears to have an active role in the formation of the biofouling layer. The particles floating above the membrane appear to be captured by the upper layer, which might also have a function of facilitating nutrient access to the lower dense layer. It should be noted again that this particular morphology could only be observed using OCT imaging as it acquires scans *in-situ* and directly in the system without affecting the sample under continuous operation. However, further detailed investigations using higher scan frequencies in the range of seconds are necessary to target the behavior observed for this particular phase/morphology.

### Multi-layer structure

**Figure 7:**
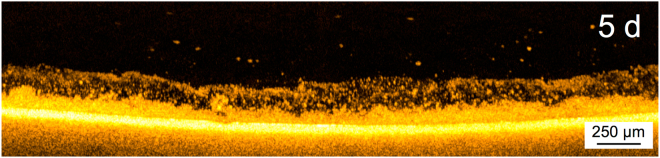
Double layered morphology observed on 5 d of GD-SMBR operation.

**Figure 8:**
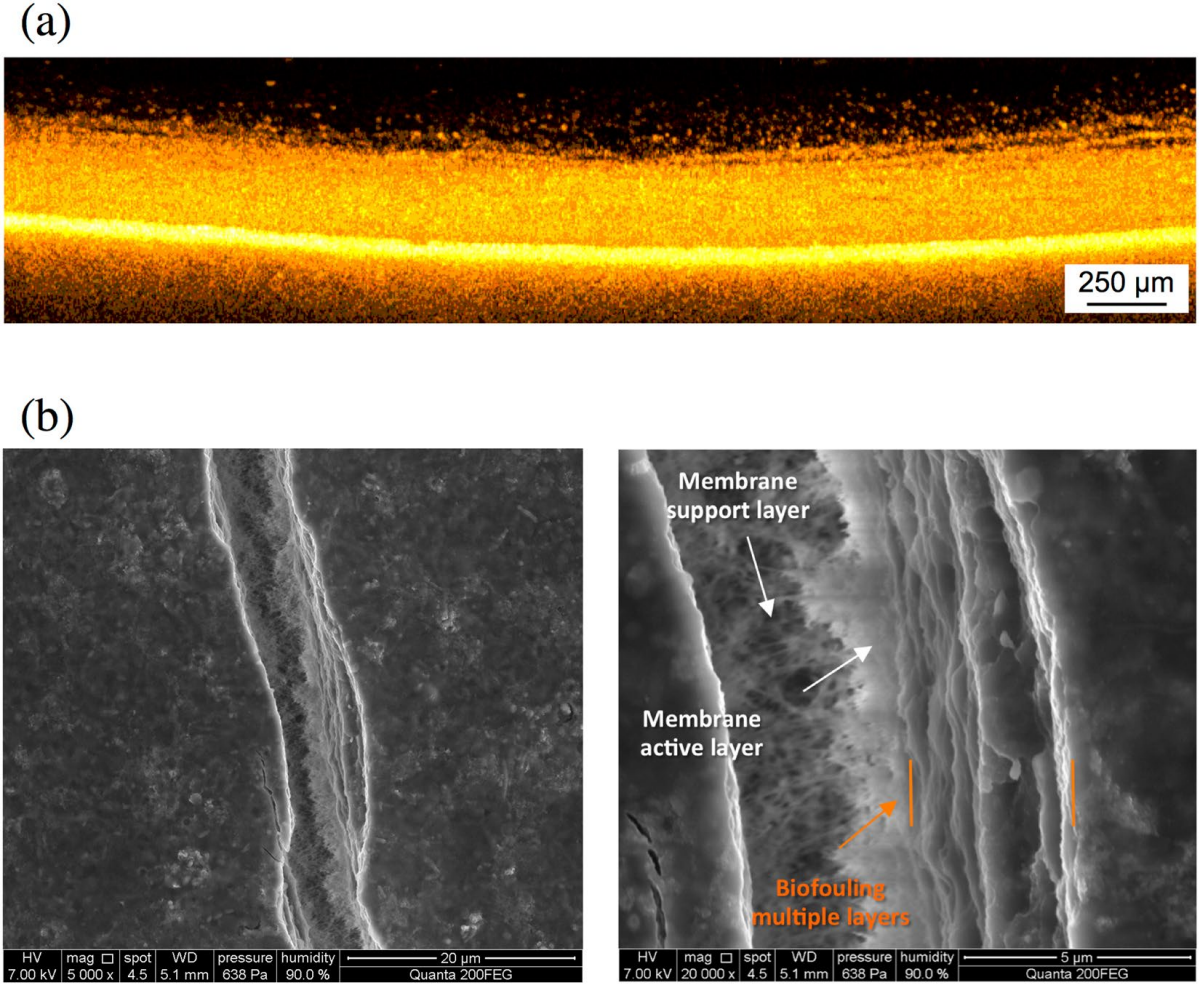
(**a**) OCT cross-sectional biofouling analysis after 42 d. It is possible to observe the presence of biofouling multilayers above the membrane. (**b**) ESEM tilted surface image of the fouling layer after 42 d. The fracture on the surface was produced by gradually decreasing the humidity in the chamber.

In the second experiment (Experiment 2), the biofouling formation on a flat sheet membrane was monitored for a period of 42 d acquiring daily scans. In this experiment the organic load was increased and more activated sludge added compared to the first experiment to enhance the biofouling formation on the membrane surface. The biofouling structure changed over time showing different morphologies (Supplementary Video 3). Biomass could be observed on the membrane from the first day. During the first 7 d of operation (Fig. 7), the biofouling displayed the same morphology observed in Experiment 1 operated with a lower organic load. However, the upper layer was observed to start collapsing from 8 d. Video analysis indicates that there is a presence of fouling layers compression by the amount of biomass that is deposited over time. The “steady state” biofouling layer formed after several days of operation appears to be formed by multiple layers (Fig. 8a). This may be due to the continuous formation of the upper layers that compress the layers closer to the membrane surface (Fig. S1). Recently, a study on the biomass developed in a gravity driven membrane filtration system revealed the abundance of low molecular weight (LMW) organics in the fouling layer composition^25^.

In order to confirm this structure, a membrane autopsy was performed at the end of the experiment using ESEM. This technique was chosen among the other EM techniques since ESEM allows the analysis of a wet-sample without any sample preparation thereby minimizing the risk of altering the original structure. In general, the application of EM is limited in the study of biofouling and samples containing water due to artifacts that may change the nature of the sample from the drying. In this work, the internal analysis of the fouling layer was performed with gradually decreasing the humidity in the chamber. This procedure caused the formation of fracture in the biomass layer. As shown in Fig. 8b, through the fracture formed on the surface, it is possible to observe the internal structure of the fouling layer, confirming the presence of multilayers as observed in the OCT analysis.

The results provided in this study are a clear evidence of the potential of the OCT technique enabling a time-lapse investigation in monitoring biofouling formation and its changes in membrane filtration systems. More detailed understanding of the biofouling morphology by *in-situ*, real-time, and non-destructive monitoring represents not only an accomplishment in a new level understanding of the formation mechanisms in biofouling. Observing the dynamics and evolution of changes in the morphology also gives a better insight of possible effects biofouling may have on membrane performance. This knowledge may further help to investigate and propose alternative strategies to mitigate and control biomass deposition and biofouling in real filtration systems. In industrial applications, such as the production of drinking water or the water reclamation, a simultaneous measurement of both biofilm structure by OCT and local hydrodynamic parameters is paramount for the understanding of transient biofilm dynamics. In addition, the OCT can be utilized for monitoring of particle deposition and fouling formation.

Since the fouling is a dynamic process, the monitoring of fouling development under continuous operation is highly recommended to gain a better (or real) understanding of biofouling behavior. In addition, OCT could further improve the fouling mitigation strategies as well as optimize the process (or design) and its operation. The approach used in this study can be extended into the evaluation of the effectiveness of different control strategies. For instance, operational parameters (i.e. aeration, relaxation and backflushing) or chemical cleaning are commonly used in membrane system to control and reduce biofouling thickness layer. Therefore, the OCT can be used as an online tool to investigate the effect of the operational parameters on the biomass deposited in the flow channel or developed on the membrane.

## Methods

A customized flat sheet type membrane module with an effective membrane area of 0.0045 m^2^ (0.09 m × 0.05 m) was submerged in a column type (polymethyl methacrylate) (PMMA) reactor (Fig. S2). The membrane module was manufactured by MemSis Turkey and employed a polysulfone (PS) ultrafiltration (UF) membrane (PHILOS, Korea) with 20 KDa of molecular weight cut-off (MWCO).

The system was operated in a gravity-driven filtration mode where the effluent (or permeate) was collected from the bottom of the tank, and more details can be found elsewhere^24, 25^. A synthetic secondary wastewater effluent (SSWE) was pumped continuously into a level regulator to keep the level of the feed water in the tank constant, resulting in a constant pressure head of 45 cm above the membrane (corresponding to a TMP of 4.5 kPa). Only a small quantity of activated sludge (4 mg of MLSS corrected from wastewater treatment plant at KAUST in Saudi Arabia) was initially added to the filtration tank to enhance the formation of a biofilm on the membrane surface. The whole reactor was covered with aluminum foil to elude the growth of algae by light exposure. The experiment was divided into two sets. The first set was conducted for 7 d with the aim to observe the initial stages of biofouling formation, and the second one was continued for 42 d to observe more long-term biofilm developments.

A SSWE was used as feed solution to grow the biofilm on the membrane. The detailed characteristics of feed water can be found elsewhere^26^. The feed solution was refreshed every 7 d. Chemical oxygen demand (COD) of the SSWE was 7.5 mg/L for the first set of experiments. In order to enhance the biofilm formation, for the second experiment the COD concentration of the feed water was increased to 15 mg/L.

The permeate flow rate was measured using a flow meter (Sensirion). The permeate flux was calculated by dividing the permeate flow by the membrane area (0.0045 m^2^). As mentioned above, the GD-SMBR was operated under constant pressure by maintaining a constant water head as shown in Fig. S2. A constant TMP of 4.5 kPa was applied to all experiments.

In this study, the OCT was employed to investigate the biofilm formation (or growth) on a membrane (submerged) in a GD-SMBR. The OCT (Thorlabs GANYMEDE spectral domain OCT system with a central wavelength of 930, Thorlabs, GmbH, Dachau, Germany) equipped with a 5X telecentric scan lens (Thorlabs LSM 03BB) was used.

The time-resolved OCT investigation was performed in three different periods of the filtration runs as given in Table S1. In the first experiment (Experiment 1), a fixed position corresponding to 5.00 mm × 1.35 (width × depth) was monitored. The scan frequency was set to 10 min for the first 42 h (from 12 h to 42 h), and then 5 min from 84 h to 96 h. The second experiment (Experiment 2) was conducted for a period of 42 d, where OCT scans were acquired daily at a fixed position corresponding to 4.00 mm × 1.35 mm.

Serial static images (i.e. video) are commonly used to depict the dynamic process of fouling formation. In this study, three videos (Supplementary Videos 1–3) of the periods monitored (Table S1) are shown in supporting information. The preprocessed OCT scans were assembled into AVI digital movie format using Fiji software.

## Electronic supplementary material

Supplementary information

Supplementary Video S1

Supplementary Video S2

Supplementary Video S3
