# Supplementary material for: Time-resolved monitoring of biofouling development on a flat sheet membrane using optical coherence tomography
Source: Sci Rep. 2017 Feb 7;7:15. doi: 10.1038/s41598-017-00051-9 (PMC5428376; doi:10.1038/s41598-017-00051-9)
Supplement: Supplementary file 1 — Supplementary information [file 41598_2017_51_MOESM1_ESM.pdf]

Supplementary Information

Title: Time-resolved monitoring of biofouling development on a flat sheet membrane using optical coherence tomography

Luca Fortunato, Sanghyun Jeong, TorOve Leiknes*

Water Desalination and Reuse Center (WDRC), Biological and Environmental Science & Engineering (BESE), King Abdullah University of Science and Technology (KAUST), Thuwal 23955-6900, Saudi Arabia

*Corresponding author: Tel. +966 12 808 2193; Email: torove.leiknes@kaust.edu.sa

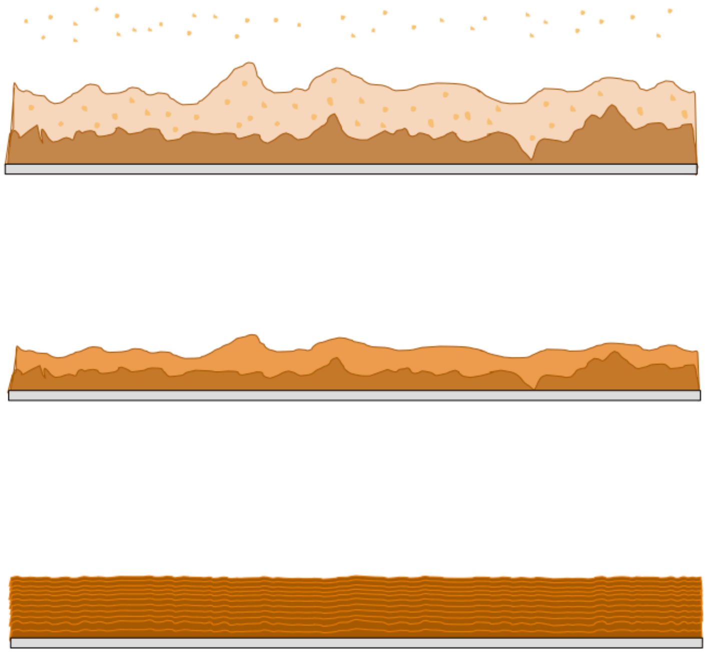

Fig. S1. Schematic drawing of biomass accumulation under gravity driven operation

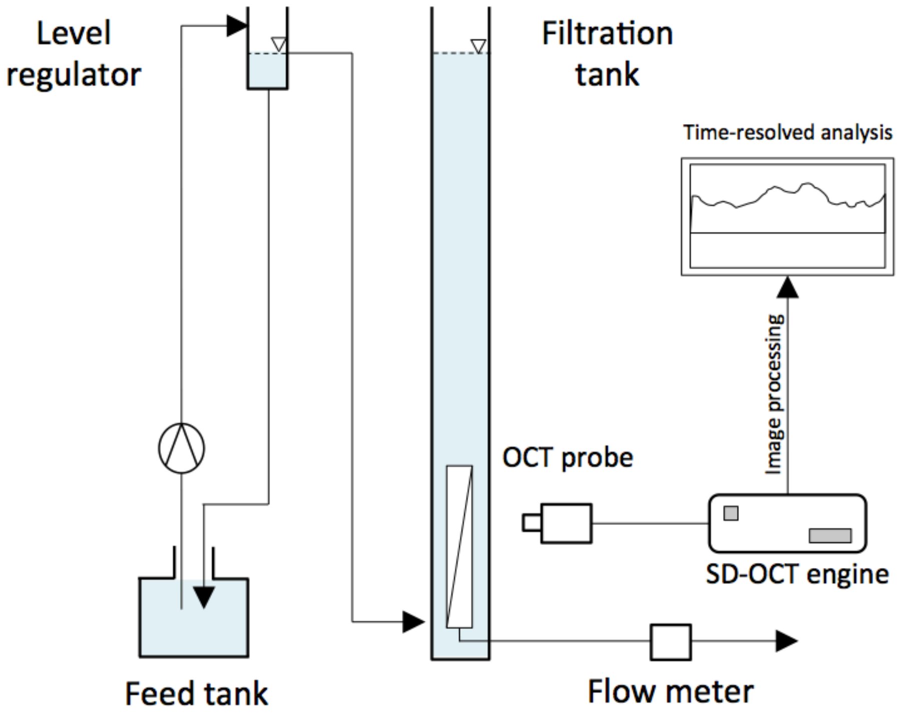

Fig. S2. Experimental setup: GD-SMBR coupled with OCT

Image Analysis

OCT scans obtained were preprocessed using FiJi software. A multi-sequence step was applied for image processing: (i) the images were filtered, (ii) contrast and brightness adjusted and (iii) the images were thresholded. Afterwards, a false scale color was applied only for visualization purpose.

Physical proprieties of the fouling layer observed were then calculated from the binarized images using customized MATLAB code that detects the 1-dimensional fouling layer interface above membrane. Each pixel corresponds to a certain length related to the acquisition setting, lateral resolution and axial resolution of 5.0 µm and 2.7 µm in air (2.1 µm in water), respectively.

The fouling coverage on the membrane (*C*) was calculated by dividing the number of pixels of the 1-dimensional layer above the membrane containing biofouling (B_i) by the total number of pixels of the 1-dimensional line that constitutes the membrane (*N*), as shown in (**Eq. 1**).

$$C = \frac{1}{N}\sum_{i=1}^{n} B_i \qquad (1)$$

The coverage was then expresses as percentage of membrane surface (C%) covered by the biofouling layer (**Eq. 2**).

$$C(\%) = C * 100 \qquad (2)$$

The mean biofilm layer thickness (Z in μm) was calculated by measuring the number of pixels from the membrane to the top layer (**Eq. 3**).

$$Z = \frac{1}{N}\sum_{i=1}^{n} Z_i \qquad (3)$$

Membrane coverage

The OCT is able to detect only a relatively thick layer deposited on a certain surface. In this work, the axial resolution in water of each scans corresponds to 2.1 μm (height of a single pixel). Different threshold values of fouled pixels (from 3 to 7 pixels) corresponding to the height of the fouling layer above the membrane were assigned to assess the percentage of membrane coverage (**Fig. S3**). The tested values presented a similar trend. The membrane coverage calculated for all the threshold values resulted to be linearly correlated (with a R^2>0.985) with the flux decrease in the period from 13 to 22 h. The value of 5 pixels height corresponding to a physical height of 10.5 μm was used to calculate the membrane coverage (**Fig. 3a**).

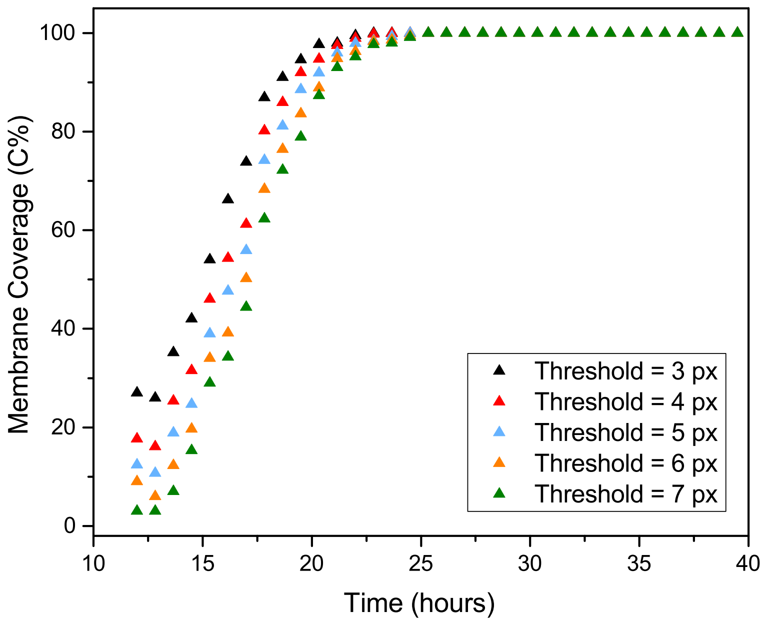

Fig. S3. Membrane coverage for different threshold pixel values for fouled biomass in the function of filtration time.

Independent Experiments

The correlation between the biomass development and the flux decrease during early stage development was validated by independent experiments. A synthetic wastewater with a similar chemical composition with a COD of 6.7±0.1 mg/L was used as feed water. The correlation between the membrane coverage and biomass thickness with flux decrease at early stage was confirmed as shown in **Fig. S4**.

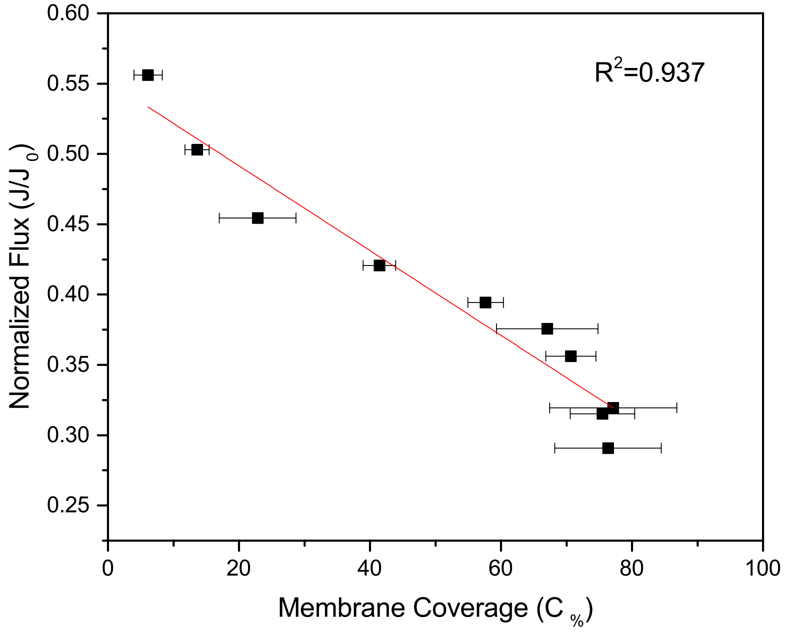

0.60
0.55
0.50
0.45
0.40
0.35
0.30
0.25
0
20
40
60
80
100
Normalized Flux (J/J 0)
Membrane Coverage (C %)
R2=0.937

(a)

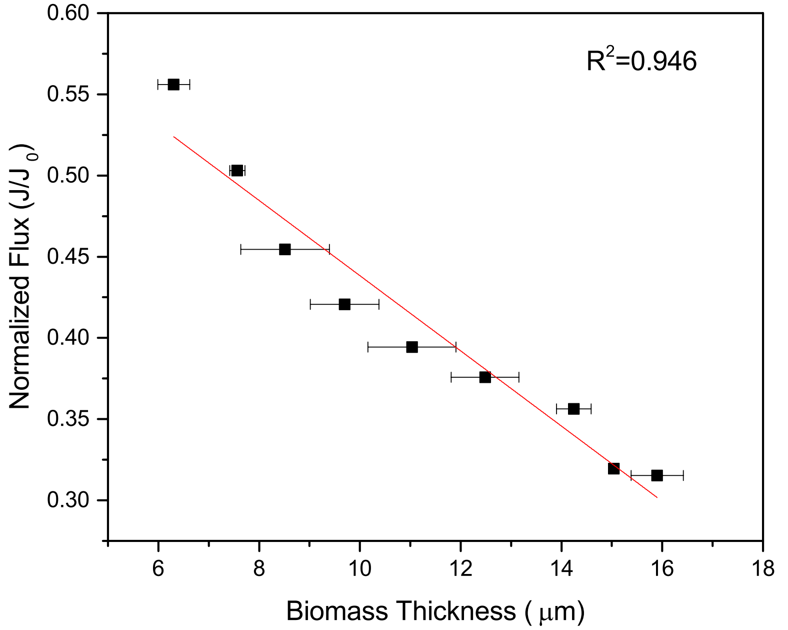

0.60
0.55
0.50
0.45
0.40
0.35
0.30
6
8
10
12
14
16
18
Normalized Flux (J/J 0)
Biomass Thickness (μm)
R2=0.946

(b)

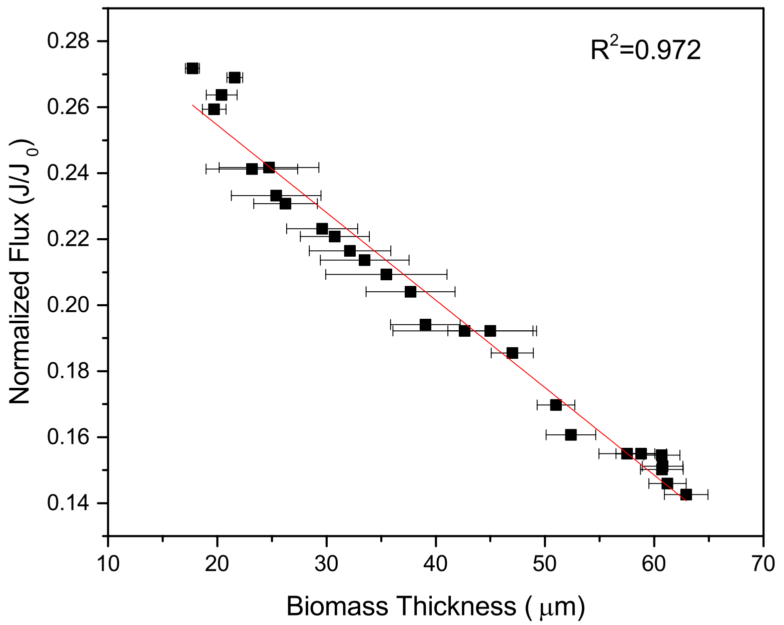

(c)

Fig. S4. a) Correlation between membrane coverage and permeate flux in the phase I, b) Correlation between average biofouling layer thickness and permeate flux in the phase I, and c) Correlation between average biofouling layer thickness and permeate flux in the phase II.

ESEM

FEI Quanta 200FEG SEM equipped with a cold stage and variable chamber pressure was used to characterize the biomass deposited on the membrane surface in a hydrated condition. Small pieces of the fouled membrane coupon were mounted flat onto an aluminum stub using thin aluminum tape. The stub was then mounted on a cold stage set at 2.0 °C. To maintain the sample in hydrated state, the SEM chamber humidity was held

at 100%. The sample was captured at accelerating voltage of 5-10 kV and working distance of 5-6 mm with gaseous secondary electron detector (GSED).

Table S1 Detailed information of OCT scans for each video.

	Experiment	OCT scan frequency	Period of observation	Duration of observation	Frame for second video (fps)
Video S1	1	10 min	12 – 42 h	30 h	10
Video S2	1	5 min	84 – 96 h	12 h	10
Video S3	2	24 h	1 – 42 d	42 d	2

Supplementary Videos

Supplementary Video 1. Early attachment.

Supplementary Video 2. Double morphology.

Supplementary Video 3. Biomass accumulation.

Three videos (Supplementary Videos 1-3) of the periods monitored are used to depict the dynamic process of fouling formation. The preprocessed OCT scans (or serial static images) were assembled into AVI digital movie format using Fiji software.
